# The Impact of Heating Rate on the Kinetics of the Nitriding Process for 52100 Steel

**DOI:** 10.3390/ma16206708

**Published:** 2023-10-16

**Authors:** Tadeusz Frączek, Rafał Prusak, Jerzy Michalski, Zbigniew Skuza, Marzena Ogórek

**Affiliations:** 1 Department of Materials Engineering, Faculty of Production Engineering and Materials Technology, Czestochowa University of Technology, 42-201 Czestochowa, Poland; tadeusz.fraczek@pcz.pl; 2 Department of Production, Faculty of Production Engineering and Materials Technology, Czestochowa University of Technology, 42-201 Czestochowa, Poland; rafal.prusak@pcz.pl (R.P.); zbigniew.skuza@pcz.pl (Z.S.); 3 Łukasiewicz Research Network—Warsaw Institute of Technology, ul. Duchnicka 3, 01-796 Warszawa, Poland; jerzymichalski987@gmail.com

**Keywords:** gas nitriding, kinetics and efficiency of the process, nitride phases

## Abstract

The aim of this study was to determine the impact of the heating rate of steel balls made of AISI 52100 alloy steel on the kinetics and efficiency of the gas nitriding process when carried out using a chemical reactor with precise thermo-gravimetric measurements, which allowed for changes in sample mass during heating and nitriding to be monitored with an accuracy of 50 µg. In the chemical reactor, the examined alloy steel was subjected to a heating process at the selected nitriding temperature of 590 °C. Two heating variants were used: the first variant relied on heating to the nitriding temperature with different rates—1 °C per minute, 2 °C per minute, 5 °C per minute and 10 °C per minute, respectively—whereas the second variant relied on the fast—25 °C per minute—heating of treated specimens to a temperature of 475 °C, at which, the nitrogenous potential of the atmosphere promotes faster nitrogen diffusion deep into the nitrided substrate, followed by reheating up to the nitriding temperature at different rates: 1 °C per minute, 2 °C per minute, 5 °C per minute, and 10 °C per minute, respectively. To evaluate the impact of heating rate kinetics and effectiveness during nitriding on the obtained surface layer quality, we investigated the phase composition, microhardness distribution, and thickness of the obtained diffusion layers. It was found that heating to a temperature of 475 °C in the nitriding process does not significantly affect the average mass gain of a sample. Above this temperature, within the range of nitriding temperatures, the extension of time increases the sample’s mass gain. Simultaneously, it was found that the use of a constant heating rate allows for thicker nitrided layers and a greater sample hardness to be obtained. Dual-stage heating, in turn, is more effective in the context of sample mass gain per time unit.

## 1. Introduction

Dynamic modern science and technology development necessitates the use of materials with ever-increasing mechanical properties, especially fatigue strength, corrosion resistance, and wear strength in friction conditions in kinetic pairs of machine mating components or structures. These fundamental features depend on these constituted specimen surface layer properties. In modern engineering practice, the surface layers being used, along with the different chemical and phase compositions and morphologies of the microstructure phase components, are produced mainly through heat and thermo-chemical treatment processes.

Various surface engineering methods are used to shape surface layer mechanical and performance properties, may of which have experienced intensive development over the last few decades and in the previous and current century in particular. This was caused by the necessity to provide new design solutions, requiring improvements in material mechanical and performance properties to reduce energy consumption and lower operating costs while observing environmental protection regulations.

Having reviewed the existing literature (a review of which is provided in Section 2 of this paper), we can state that, currently, the fastest developing surface engineering methods include nitriding, heat and thermo-chemical treatment in vacuum, and low-temperature plasma and plasma and laser methods [1]. 

Special attention has been given to the constitution of surface layer properties through the development of the following processes:

1. Nitriding, carbon-nitriding, oxy-carbon-nitriding, boroning, and oxy-nitriding using glow discharge for the electrical activation of direct currents and pulsed plasma environments;
2. Carburizing or nitro-carburizing under reduced pressure, with pulsed carbon or nitrogen-bearing gas injection;
3. Gas nitriding and its variations (used to produce wear- and corrosion-resistant layers for steel grades that are difficult to nitride in frictional conditions).

The gas nitriding process (and its variations) is still one of the surface engineering methods that increases machine component performance and structural properties, with the most important factor being the tribological properties and the corrosion resistance thereof. This surface engineering development method is aimed towards reducing processing times and replacing dissociated ammonia with nitrogen.

The aim of this study was to determine the impact of the heating rate of steel balls made of AISI 52100 alloy steel on the kinetics and efficiency of the gas nitriding process. The authors of the article assumed that the heating rate may significantly affect the phase structure of the near-surface zone of iron nitrides. Regardless of the temperature and nitrogen potential, the heating rate has a significant impact in shaping the kinetics of the growth of the nitrided layer on steel.

## 2. Literature Review

The majority of techniques that involve material surface modification are based on processes in which, as a result of chemical reactions, surface layer structures and properties are modified [2]. Gas nitriding (GN) is thermo-chemical treatment used to improve specific properties of nitrided materials [3]. When correctly carried out, the nitriding process can lead to, among other things, improvements in surface hardness [4] and material corrosion resistance [5] and increases in wear resistance [6] with only minor dimensional changes to the nitrided components [7]. 

Nitriding significantly extends machine parts’ service lives operating under heavy loads [8]. Improving surface properties is a consequence of nitride compound layer formation on the surface above the diffusion zone in which precipitated alloy nitrides are located [9]. The nitrogenous layer consists of a combined zone, including (but not limited to) iron nitrides ε (nitride Fe_2_N_1−x_—solid nitrogen solution based on Fe_2_N nitride) and γ′ (nitride Fe_4_N_1−x_—solid nitrogen solution based on Fe_4_N nitride) and a diffusion zone with γ′ nitride precipitates. A lower portion of the ε phase promotes wear resistance improvement, while increases in this phase portion (with limited porosity) contribute to increased corrosion resistance [10]. 

Nitride layer formation in the gas nitriding process begins in so-called active centres (energetically preferred places)—e.g., at grain boundaries, inclusions, or defects [11]. In effect, the number of such centres significantly affects process kinetics (a higher nitrogen concentration in these areas contributes to faster nitride nucleation) [12]. It should be noted that besides the nitrided layer having a small surface thickness, after process termination [13], the rest of the nitrided component (its interior) remains unchanged. Nitriding, in the context of gas nitriding, occurs at the interface between NH_3_ gas and the workpiece surface, and atomic nitrogen adsorption onto the surface occurs, and nitrogen diffuses deep into the material [14]. As a result of atomic nitrogen diffusion into the steel surface, a surface layer with different properties compared to the original material is formed. Phase structure, thickness, the type of occurring phases, and consequently, the properties thereof depend both on steel grade and nitriding conditions. The basic nitriding process parameters include the following: temperature T, atmosphere nitrogenous potential *NP*, time t. 

Nitrogen potential is a parameter that determines the probability of creating nitrogen layers with a specific structure. This potential can be expressed by the following formula [15]:

$$NP=\frac{p_{NH_{3}}}{p_{H_{2}}^{3/2}}$$

**Figure 1:**
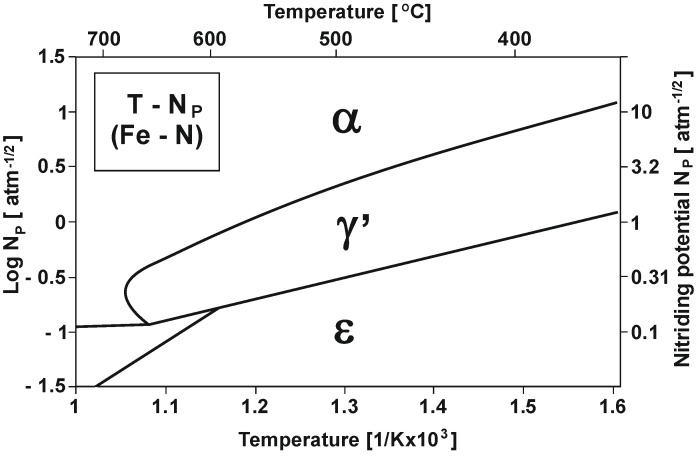
T—NP equilibrium configuration (Lehrer’s system) [15].

Figure 1 shows the varying nitrogenous potential of iron and carbon steels at different temperatures (according to Lehrer’s system) [15]. 

As Figure 1 shows, within the nitriding temperature range of 475–590 °C and at NP_α_ nitrogenous potential, a diffusion layer increase will only occur in the α-phase persistence region, and as a result, a single-zone layer will be obtained. At NP_γ′_ potential, an increase in the γ′ + α of the dual-phase layer will occur [16]. 

The triple-phase layer increases at NP_ε_ potential in the initial stage, and this increase identically proceeds the dual-phase layer increase. Another essential parameter besides nitriding potential is process temperature. As shown in the diagram in Figure 1, an increase in temperature decreases the nitriding potential boundary value at which the obtained layer takes form, which corresponds to phase stability in a given area.

Traditional gas nitriding, usually carried out in a single-component atmosphere (ammonia), is characterized by very limited control of layer gain kinetics, most frequently consisting of ε + γ′ + α zones. To obtain adequate performance properties, sometimes it is necessary to remove the iron nitrides ε + γ′ near-surface layer (which is excessively thick and brittle) via grinding. In addition, the formation of the ε + γ′ + α layer requires a much higher nitriding potential than that for the γ′ + α layer formation. This necessitates a higher ammonia flow rate and, as a result, a higher consumption of ammonia. From an economic point of view, the increased demand for gas and the subsequent removal of ε + γ′ compounds is very disadvantageous [17]. 

GN kinetics depends on process parameters, i.e., temperature, time and pressure, and the specifics of the surface to be treated [18] as well. Standard GN processes are carried out in a temperature range of 500–590 °C, take anywhere from several hours up to a dozen hours while requiring high energy consumption [19], and are one of the more energy-consuming processes in the manufacturing industry [20]. Under industrial conditions, it is difficult to achieve satisfactory efficiency and productivity levels [3]. A desire to achieve greater process efficiency is the driving force behind the numerous studies accelerating process kinetics and process cost reduction [21,22]. The nitrided layer thickness depends on the conditions of conducted process, the properties of the material being nitride, and the reactions occurring on the material surface and the atom diffusion deep into material [23]. Reaction acceleration occurring on the treated material surface was achieved, among other things, through using component surface pre-treatment prior to basic nitriding process in [24,25]. Surface nanocrystalline layer formation allows process kinetics to be improved and may contribute to increasing the hardness and wear resistance of the layer formed, based on the results of conventional nitriding [26]. The gas pressure applied during nitriding process is of great importance for nitriding processes, as it affects nitrogen potential [27]. An increase in nitriding pressure may contribute to shortened process times (e.g., increasing pressure from 1 to 6 atm within 5 h, effect similar to 50 h process [19]), and one study in the literature reported a notable nitrided layer thickness increase (pressure increasing from 1 to 5 atm, at the same process time, resulted in the nitrided layer thickness increasing from 210 μm to 1100 μm [18]). Simultaneously, reducing pressure whilst using other appropriate process parameters may lead to an improvement in surface strength and corrosion resistance while maintaining a nitrided layer thickness similar to that obtained in the process carried out at pressure of 1 atm [5]. An increase in process temperature raises its kinetics, resulting in shorter process proceeding times [28]. Lowering the process temperature level is likely to bring potential economic advantages; however, unfortunately, this translates into negative process effects such as a decrease in material fatigue limit [6]. 

An alternative for traditional gas nitriding is using controlled, in most cases, dual-stage process. The aim of first stage is to saturate the surface layer with nitrogen and form an iron nitride film layer. This is realized at high nitrogen potentials and lower temperatures. The second stage occurs at higher temperatures and lower nitriding potentials and is caused by the internal nitriding zone expanding. In this case, the iron nitride layer and nitriding atmosphere [17] are the nitrogen sources. A controlled process, supported by appropriate technological developments, influences, through precise nitriding parameter selection (T, NP, t), the nitrided layer phase structure and shape zone thickness therein. In effect, less gas and energy is consumed, and product surface grinding is avoided.

The aim of this study was to determine the preliminary influence of the heating rate of steel balls made from 52100 steel on the kinetics and efficiency of the gas nitriding process through an investigation involving the use of a chemical reactor with precision thermo-gravimetric measurements to enable the monitoring of accurate sample mass change during heating and nitriding with an accuracy of 50 µg.

## 3. Research and Methodology

**Figure 2:**
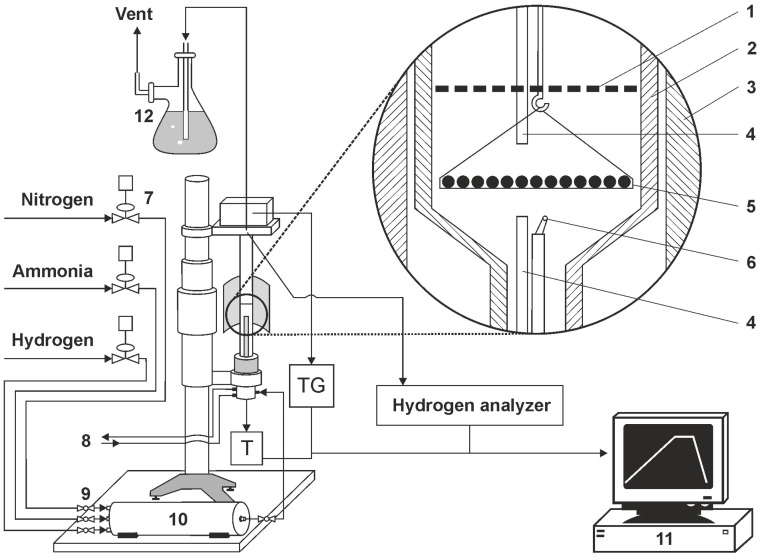
Diagram of a test bench with a thermocouple (1—shutter, 2—reactor wall, 3—reactor furnace, 4—gas phase sampling point, 5—sample holder with single layer of samples, 6—thermocouple, 7—electronic flowmeters, 8—cooling water (closed circuit), 9—ball valve, 10—gas mixer, 11—process control computer, 12—scrubber (distilled water).

Within the research framework used, AISI 52100 alloy steel was subjected to a heating process at a selected nitriding temperature of 590 °C. The process was carried out in a chemical reactor with the high-precision thermo-gravimetric measurement device, type SRT-10, (so-called thermoweight, Figure 2), enabling the monitoring of sample weight change during nitriding and annealing with a resolution of 50 µg. The software-controlled thermo-gravimetric measurement reactor is designed to perform simultaneous measurements of temperature in the active space of the chemical reactor and temperature and solid substance sample mass changes that are surrounded by gas or gas mixtures under heating up conditions, temperature stabilisation, or reactor active space cooling.

The programmable measurement interface enables the recording of measurement cycle results of mass and temperature changes. The maximum flow rate of NH_3_ is 200 ml/min, and the flow rate of other gas media is at a similar level. The maximum temperature in the working chamber is 1000 °C. A diagram of the test stand with a thermocouple is presented in Figure 2.

Two heating up variants were used:

- The first variant centred around increasing the nitriding temperature at the following rates of: 1 °C/min, 2 °C/min, 5 °C/min, and 10 °C/min.
- The second variant relied on the rapid heating up of workpieces—25 °C/min to a temperature of 475 °C, in which the atmospheric nitrogenous potential promoted faster nitrogen diffusion deep into the nitrided substrate with subsequent reheating to nitriding temperature at different rates as follows: 1 °C/min, 2 °C/min, 5 °C/min and 10 °C /min, respectively.

In addition, a different atmosphere composition of 190 mL/min NH_3_ + 10 mL/min H_2_ was used for three processes (e, h, and k). We applied the presented heating variants to investigate whether changes in heating rate affect iron nitride formation kinetics in the 52100 steel under study and whether changes in heating rate affect the thickness and hardness of the obtained nitrided layers. 

The alloy steel AISI 52100 was subjected to the nitriding processes. AISI 52100 steel, in addition to carbon and ordinary admixtures, contains 1.5 wt. % chromium. Chromium is an element with a high affinity for nitrogen. The chemical composition of the steel and the diameters of the samples in the form of a sphere are given in Table 1. The parameters of the nitriding processes are given in Table 2.

Hardness distribution measurements of nitrided layers were executed using Vickers’s method on a semi-automatic FM 7 hardness tester from FUTURE-TECH at a load of 100 G (980.7 mN).

The phase composition of the iron nitride layer on the samples was determined via X-ray diffraction using the Seifert 3003TT X-ray diffractometer (Seifert, Ahrensburg, Germany) using KαCo radiation and symmetrical measurement geometry. The measurement parameters were as follows: voltage 30 kV, current 40 mA, step 2θ 0.05°, and counting time 5 s. The range of recorded diffraction angles was 40–58°, and this range included the main characteristic lines from iron nitrides γ′ and ε and from the steel substrate, according to patterns from the PDF4+ diffractometric database.

The iron nitride layer thickness assessment method that we used and refer to in this paper has been described elsewhere [25].

## 4. Results and Discussion

**Figure 3:**
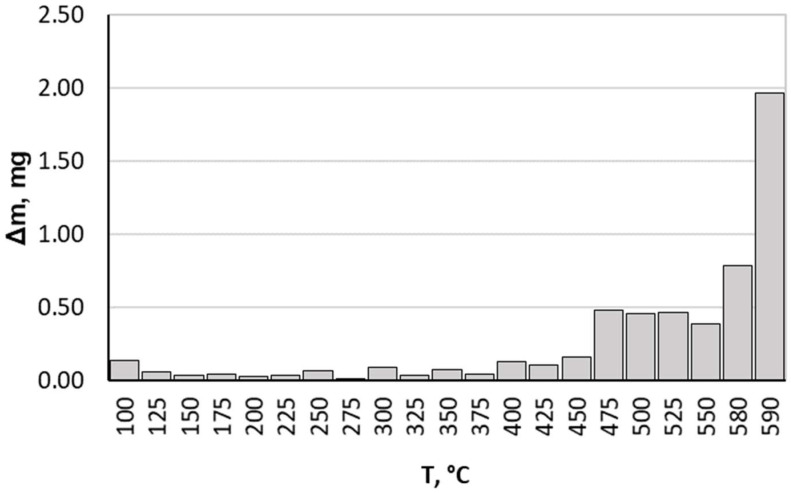
Average sample mass change during process proceeding.

When analysing the results we obtained, it should be noted that up to a temperature of 475 °C, the nitrided component average mass gain was small, achieving a value of 0.162 mg at this temperature for all heating rates. Above this temperature, more rapid nitrided sample mass gain occurred (Figure 3). This is due to greater ammonia dissociation intensity affecting nitrogenous potential change, which in turn translates into greater nitrogen diffusion deep into the nitrided substrate. Taking this fact into consideration, only data on processes carried out within a temperature range of 475–590 °C are presented and analysed in this paper.

### 4.1. Formed Layer Thickness and Composition Investigation

**Figure 4:**
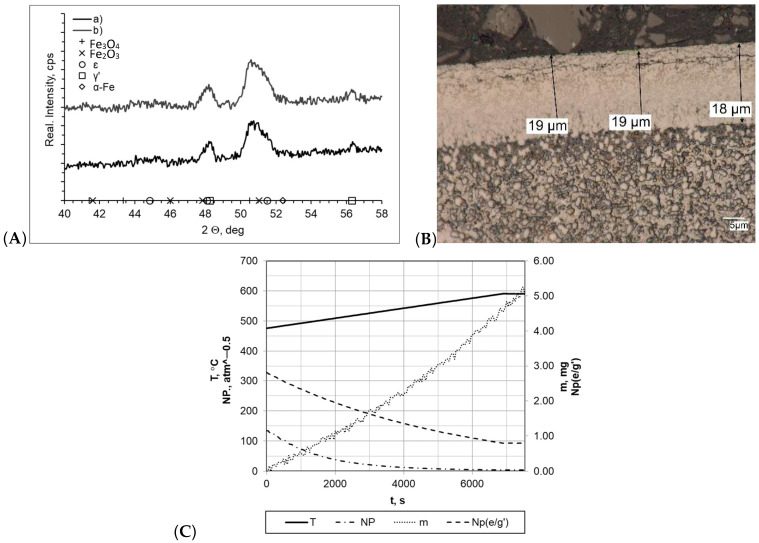
(**A**) X-ray analysis results for samples a and b; (**B**) microscopic analysis results and process proceeding data for sample a. (**C**) Process proceeding characteristics (T—temperature, NP (nitrogenous potential, m—mass gain, NP (e/g’)—boundary potential e/g’ as temperature function) for sample a.

When both samples were heated at a rate of 1 °C/min (within temperature range 475–590 °C), the layers formed during nitriding process were of uniform thickness, and the boundary between the layer and the substrate was clearly visible (Figure 4). In both cases, the nitride layer thickness was very similar and was g_mp_ = 19 μm for the processes carried out with a constant heating rate (sample b) and g_mp_ = 18 μm for processes carried out with two heating rates (sample a). In the diffractograms, we identified lines characteristic for the γ′ layer within the range of the 2θ angles examined. Among all of the processes, the nitrogenous potential was at a high level, and in both cases, the minimum value thereof was approximately NP = 3.45–3.65 atm^−0.5^. In both cases, the sample mass gain was almost identical and was within a range of Δm = 5.05–5.07 mg. However, it should be mentioned that significant differences in process times significantly affected process efficiency (mass gain per time unit was 0.0268 mg/min and 0.0084 mg/min for samples a and b, respectively).

**Figure 5:**
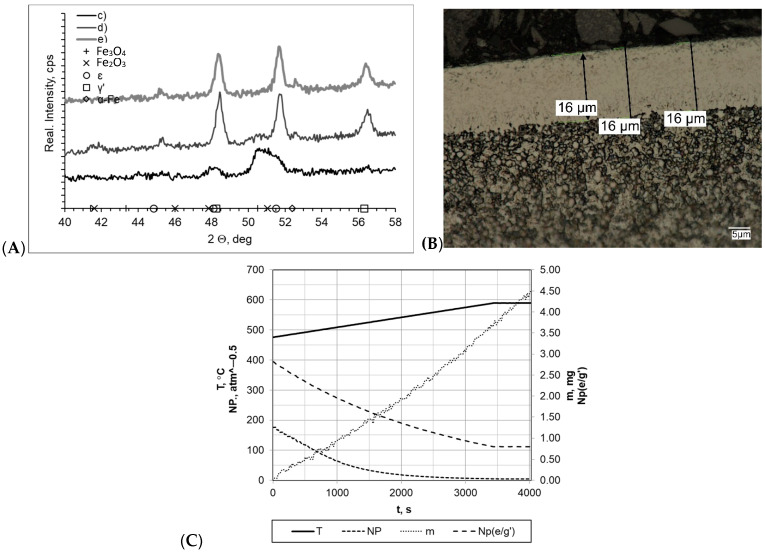
(**A**) X-ray analysis results for samples c, d, and e; (**B**) microscopic analysis results and process proceeding data for sample c; (**C**) process proceeding characteristics (T—temperature, NP—nitrogenous potential, m—mass gain, NP(e/g’)—boundary potential e/g’ as temperature function) for sample c.

Detailed process proceeding data for the samples heated within a temperature range of 475–590 °C at rate of 2 °C/min are shown in Figure 5. In all cases, layers of uniform thickness with a clear boundary between the layer and the substrate were formed, and the layer thicknesses varied between each other. The greatest layer thickness was found for samples c and e, for which the process was carried out with a constant heating rate (g_mp_ = 16 μm and g_mp_ = 15 μm, respectively) and H_2_ addition at 10 ml/min contributed to forming a thinner layer. Regarding the results of the process carried out with double-stage heating, the formed layer had a significantly smaller thickness (g_mp_ = 12 μm). These differences were reflected in the sample mass change magnitude. The greatest changes were recorded for samples c and e, and they were approximately Δm = 4.55 mg. The mass change for sample d was definitely smaller, amounting to Δm = 2.42 mg. The mass change magnitude values for samples c, e, and d were 0.0135 mg/min and 0.0229 mg/min, respectively. There were marked differences in the nitrogenous potential values in all processes. The highest potential occurred in process c, wherein, at 475 °C, it was NP = 177.64 (minimum potential value—at 590 °C—was NP = 4.20 atm^−0.5^). In other cases, it was definitely lower, and for the samples d and e, it amounted to NP = 17.87 atm^−0.5^ to NP = 0.37 atm^−0.5^ and NP = 0.016 atm^−0.5^ to NP = 0, 15 atm^−0.5^, respectively. These differences were reflected in diffractograms, which, for samples d and e, showed the presence of a double-phase layer consisting of γ′ and ε iron nitrides, whereas for sample c, only lines characteristic for the γ′ phase were found. Lines characteristic of the α-Fe substrate were also identified in the diffractograms.

**Figure 6:**
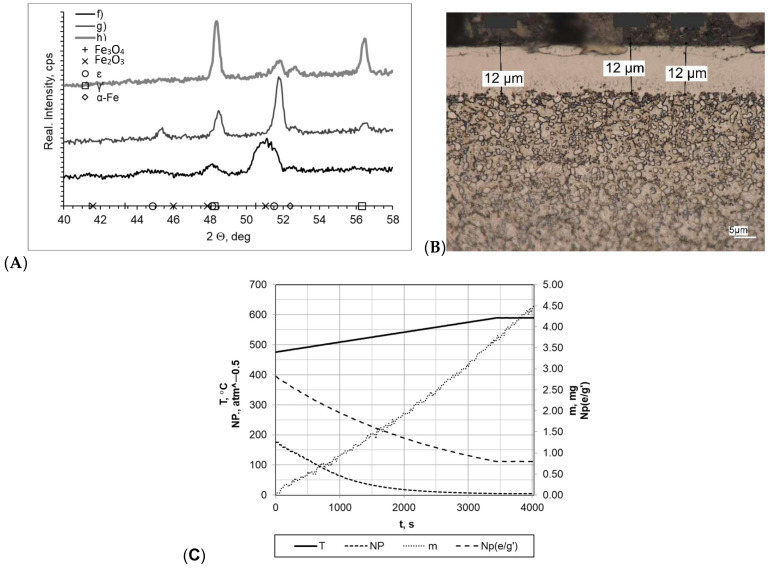
(**A**) X-ray analysis results for samples f, g, and h; (**B**) microscopic analysis results and process proceeding data for sample g; (**C**) process proceeding characteristics (T—temperature, NP—nitrogenous potential, m—mass gain, NP(e/g’)—boundary potential e/g’ as temperature function) for sample g.

Subsequently, the tested samples were heated within a temperature range of 475–590 °C at a rate of 5 °C/min (Figure 6). All of the formed layers were double-phase layers with uniform thickness and a clear boundary between the layer and the substrate. The greatest layer thickness was obtained for sample f (g_mp_ = 14 μm); in other cases, the layer thickness was smaller (g_mp_ = 12 μm for sample h and g_mp_ = 11 μm for sample g). Nitrogenous potential also varied in individual processes, and the greatest one was for sample f (NP = 5.60 atm^−0.5^ at 475 °C and NP = 5.60 atm^−0.5^ at 590 °C). In the other cases, it was NP = 0.63 atm^−0.5^ at temperature of 475 °C and Np = 0.48 atm^−0.5^ at temperature of 590 °C (sample g) and Np = 0.48 atm^−0.5^ at temperature of 475 °C and NP = 0.21 atm^−0.5^ at temperature of 590 °C (sample h). Diffractograms allowed us to conclude that there are differences between the individual samples. The layers formed, in the case of g and h, were double-phase layers, and lines characteristic for γ′ and έ iron nitrides occurring within the range of the 2θ angles examined were recorded. Lines characteristic for the α-Fe substrate were also identified in the diffractograms. The sample mass gain values were very similar overall; they are as follows: sample f Δm = 2.30 mg, sample g Δm = 2.25 mg, sample h Δm = 2.15 mg. The differences in process implementation time were reflected in the values recorded for process efficiency. The values for the mass gain over time for samples f, g, and h were 0.0295 mg/min, 0.0553 mg/min, and 0.0266 mg/min, respectively.

**Figure 7:**
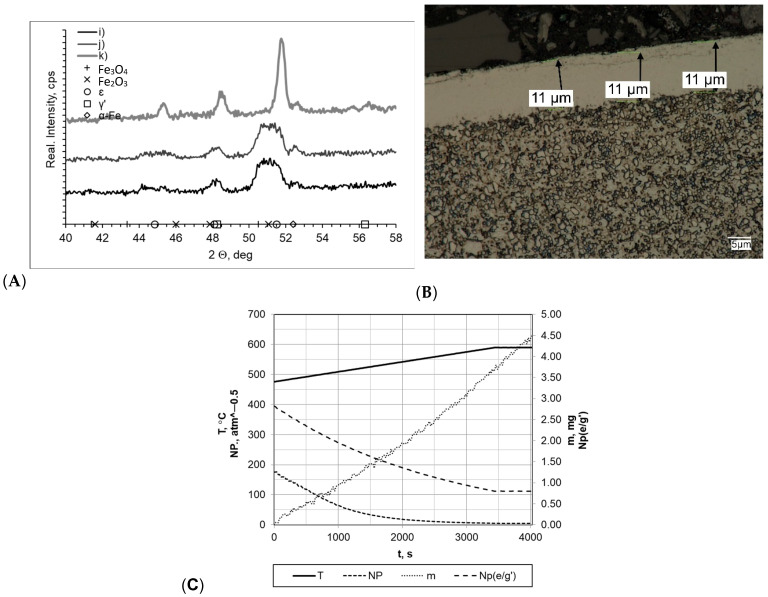
(**A**) X-ray analysis results for samples i, j, and k; (**B**) process proceeding characteristics (T—temperature, NP—nitrogenous potential, m—mass gain, NP(e/g’)—boundary potential e/g’ as temperature function) and (**C**) microscopic analysis results and process proceeding data for sample i.

In all processes carried out at a rate of 10 °C/min (within a temperature range of 475–590 °C), the layers formed had very similar thickness values (g_mp_ = 12 μm for sample k and g_mp_ = 11 μm for samples i and j; Figure 7). In every case, it was observed that the layers had uniform thickness and a clear boundary presence between the substrate and the layer. Slight layer thickness differences were reflected in the mass change magnitude, which fluctuated between Δm = 3.90 mg (sample i) and Δm = 3.50 mg (sample j). However, it should be mentioned that essential differences in process time significantly affected process efficiency (the mass gain per time unit values for samples i, j, and k were 0.0420 mg/min, 0.0538 mg/min and 0.0388 mg/min, respectively). Diffractograms allowed us to observe the differences between sample k and samples i and j. Regarding sample k, its diffractogram showed the presence of iron nitrides γ′ and έ. For samples i and j, their nitrogenous potential values were at a high level throughout the entire nitriding process time, amounting to Np = 5.09–194.66 atm^−0.5^ and Np = 8.59–483.56 atm^−0.5^, respectively. In terms of sample k, its potential was definitely lower, amounting to Np = 0.59 atm^−0.5^ at 475 °C and Np = 0.47 atm^−0.5^ at 590 °C.

A summary of the test results for the individual processes is presented in Table 3. Process proceeding decreases resulted in decreases in the thicknesses of the obtained layers. Simultaneously, it can be observed that, in each of nitriding the variants used, the thickest layer was obtained after the process conducted at a constant speed (samples c, f, and i). Using a modified atmosphere consisting of 190 mL/min NH_3_ + 10 mL/min H_2_ contributed to the attainment of thinner layers compared to those derived from the use f an atmosphere consisting of only NH_3_.

### 4.2. Sample Hardness Testing following the Nitriding Processes

Microhardness distribution was measured at a load of 980.7 mN (100 G), with the first measurement located 25 µm from the surface. Subsequent measurements were made with measuring steps of 50 µm up to a distance of 500 µm from the surface, and subsequent measurements were made every 100 µm until the hardness value corresponding to the hardness of the material core was reached.

**Figure 8:**
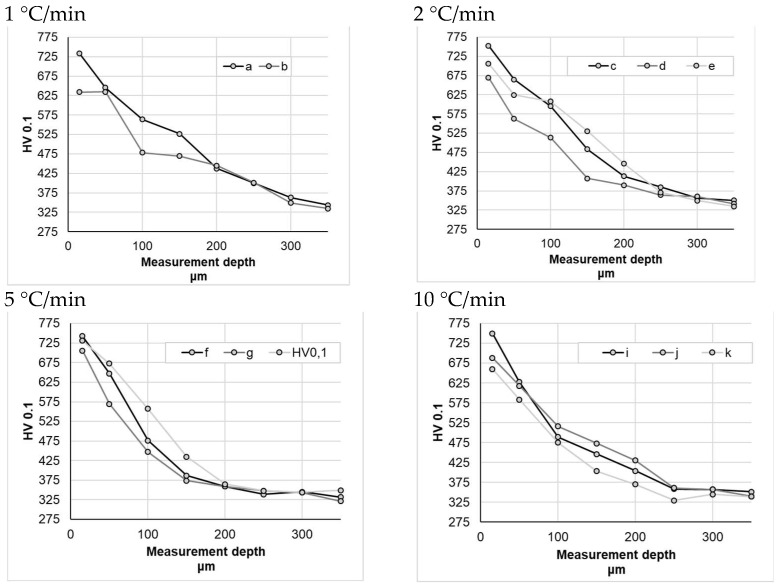
Hardness measurement results for different heating rates at temperatures above 475 °C.

Sample hardness testing did not reveal significant differences between the individual nitriding variants (Figure 8), whereas the mild hardness distribution profile in the obtained nitrided layers is noticeable. For the samples nitrided at a heating rate of 1 °C/min—within a measurement range from 0 to 200 μm—sample a (subjected to heating at a constant heating rate) had an average hardness that was approximately 10% higher than that of the sample b. For the samples nitrided at a heating rate of 2 °C/min, the difference between the sample nitrided at a constant heating rate (c) and the sample nitrided at variable heating rates (d) was even greater, amounting to approximately 15% (within measurement range from 0 to 200 μm). For the samples nitrided at higher nitriding rates, the differences were smaller, ranging from 1% (for a heating rate of 10 °C/min) to 5% (for a heating rate of 5 °C/min) on average.

## 5. Conclusions

Nitriding processes may, in effect, result in the formation of nitrided layers containing basic zones: nitride zone ɛ and ɛ + γ′, γ′. Just below the nitride zone, there is a diffusion zone. The layer of nitride compounds formed on the sample surface is responsible for improving wear resistance, galling (tribological properties), and corrosion resistance. The diffusion layer is responsible for improving fatigue strength and acts as the substrate for the hard compound film layer, shaping optimum stress distribution characteristics throughout the entire layer section. Modern gas nitriding facilities allow for the resulting nitrided layer structure to be precisely controlled and shaped. Controlling and regulating the process using technically advanced atmosphere analysers, mass flow controllers, and homogeneous atmosphere circulation ensures the optimum layer for the actual component operating conditions to be obtained.

The effectiveness of any activity is measured by the ratio of effects to expenses. In the case of gas nitriding, we would like to obtain appropriate thicknesses and nitrided component surface layer morphologies. Such a process is defined primarily by temperature and working gas flow intensity. Both of these factors are a function of time and determine the final economic effect of nitriding. 

Based on the cases analysed for this paper, we draw the following conclusions:

- A heating rate up to 475 °C does not significantly affect sample average mass gain.
- It should be stated that up to a temperature of 475 °C, the nitrided sample average mass gain is small, reaching a value of 0.162 mg at this temperature for all heating rates thereof. Above this temperature (but still within the nitriding temperature range), the extension of time increases sample mass gain. Therefore, it seems to be logical to reduce the sample heating period in the reaction chamber to 475 °C.
- Specified gas precision dosing and optimum heating rate selection (temperature increase rate and electric current consumption; sample mass and heat capacity) should constitute the first step towards process cost limiting. However, it should be mentioned that some studies have shown that despite the better process efficiency (greater sample mass gain per time unit), using double-stage heating contributes to thinner nitrided layers and lower sample hardness in the near-surface layers (especially for lower heating rates).
- The reaction gas composition and the flow rate thereof in individual stages of the process may be indicative of the nitrogenous potential, which seems to be a separate issue. According to Lehrer’s system, this parameter determines individual phase durability areas and, to some extent, affects the kinetics of the entire process.
- Using a modified atmosphere of 190 mL/min NH_3_ + 10 mL/min H_2_ in the nitriding process resulted in a slight reduction in nitride layer thickness, whereas the hardness of our obtained samples (with respect to the samples nitrided in 200 mL/min in NH_3_ atmosphere) varied—within a measurement range of 0 to 200 μm—from + 6% for the heating up variant with a heating rate of 5 °C/min to 9% for the heating up variant with a heating rate of 10 °C/min. For a heating rate of 2 °C/min, the average hardness difference was close to 0. Taking into consideration these results, it is difficult at this stage to explicitly demonstrate the advantages or disadvantages of using such a modified atmosphere. In order to achieve that aim, additional research is required.

The main achievement of this study is the fact that it demonstrates that the heating rate significantly affects the kinetics of the nitriding process, thereby affecting the phase structure of the near-surface zone of iron nitrides.

This study had some specific features and limitations that should be mentioned. Firstly, the experimental methodology we used resulted in high nitrogenous potential values in the initial process phases. The potential values decreased as the process continued and the temperature was raised. The use of so-called thermobalances limited the weight of the nitrided elements to a maximum of 10 g, while the use of samples in the form of balls with a diameter of 3 mm limited the possible weight of the samples (shape, empty spaces). An interesting aspect to explore in further research is the possibility of nitriding uniform elements of greater mass. Under industrial conditions, the weight and size of the heating chamber and samples will significantly affect (due to heat capacity) heating time and, consequently, process proceeding (meaning that processes carried out while reaching nitriding temperature must be taken into account).

## Figures and Tables

**Table 1:** Chemical composition of steel used in the tests.

Grade Steel	ϕ	Element Content in wt. %						
	(mm)	C	Mn	Si	P	S	Ni	Cr
AISI 52100	3	1.0	0.4	0.3	0.02	0.02	-	1.5

**Table 2:** The basic parameters of the nitriding processes.

Sample No.	T [°C]	Process Type *	t [min] Whole	t [min] from 475 °C	ΔT [°C]	NH_3_, [mL/min]
a	590	2	165.2	125	1	200
b		1	592.4			
c	590	1	312.4	67.5	2	200
d		2	107.7			
e		1	312.4			190
f	590	1	144.4	33	5	200
g		2	73.2			
h		1	144.4			190
i	590	1	88.4	21.5	10	200
j		2	61.7			
k		1	88.4			190

* 1. Single-stage heating at a constant rate of ΔT, 2. Double-stage heating at a rate of 25 °C/min to 475 °C and at rate ΔT to T temperature.

**Table 3:** Summary of test results.

Sample	Δm_t_ [mg]	Δm_c_ [mg]	g_mp_min [μm]	g_mp_max [μm]
a	0.0084	5.05	18	19
b	0.0268	5.07	17	18
c	0.0135	4.50	16	16
d	0.0229	2.42	12	12
e	0.0144	4.55	15	15
f	0.0295	3.95	13	14
g	0.0558	3.95	10	11
h	0.0266	3.95	11	12
i	0.0420	3.90	11	11
j	0.0538	3.50	10	11
k	0.0388	3.60	11	12

Key for table: Δmt—mass gain per time unit, Δmc—total mass gain, g_mpmin_—minimum measured layer thickness, g_mpmax_—maximum measured layer thickness.

## Data Availability

The data can be found in Czestochowa University of Technology.
